# Resistance to Sliding in Clear and Metallic Damon 3 and Conventional Edgewise Brackets: an *In vitro *Study

**Published:** 2015-03

**Authors:** Mohammad Karim Soltani, Farzaneh Golfeshan, Yoones Alizadeh, Jabraiel Mehrzad

**Affiliations:** 1 Dept. of Orthodontics, School of Dentistry, Hamadan University of Medical Sciences, Hamedan, Iran;; 2 Dept. of Mechanical Engineering, School of Mechanical Engineering, Amirkabir University of Technology, Tehran, Iran;

**Keywords:** Friction, Self-ligating Brackets, Sliding Technique

## Abstract

**Statement of the Problem:**

Frictional forces are considered as important counterforce to orthodontic tooth movement. It is claimed that self-ligating brackets reduce the frictional forces.

**Purpose:**

The aim of this study was to compare the resistance to sliding in metallic and clear Damon brackets with the conventional brackets in a wet condition.

**Materials and Method:**

The samples included 4 types of brackets; metallic and clear Damon brackets and metallic and clear conventional brackets (10 brackets in each group). In this study, stainless steel wires sized 0.019×0.025 were employed and the operator’s saliva was used to simulate the conditions of oral cavity. The tidy-modified design was used for simulation of sliding movement. The resistance to sliding and static frictional forces was measured by employing Testometric machine and load cell.

**Results:**

The mean (±SD) of resistance to sliding was 194.88 (±26.65) and 226.62 (±39.9) g in the esthetic and metallic Damon brackets, while these values were 187.81(±27.84) and 191.17(±66.68) g for the clear and metallic conventional brackets, respectively. Static frictional forces were 206.4(±42.45) and 210.38(±15.89) g in the esthetic and metallic Damon brackets and 220.63(±49.29) and 215.13(±62.38) g in the clear and metallic conventional brackets. According to two-way ANOVA, no significant difference was observed between the two bracket materials (clear and metal) and the two types of bracket (self-ligating versus conventional) regarding resistance to sliding (*p*= 0.17 and *p*= 0.23, respectively) and static frictional forces (*p*= 0.55 and *p*= 0.96, respectively).

**Conclusion:**

Neither the type of bracket materials nor their type of ligation made difference in resistance to sliding and static friction.

## Introduction

Sliding a tooth along an arch wire is a very common orthodontic procedure to translate tooth, especially during the closure of spaces in extraction cases and correction of dental irregularities.[1] The advantages of this technique are shorter clinical treatment time, more patient convenience, and better controlling of three dimensional tooth movements.[2] On the other hand, one of the disadvantages of this system is the frictional forces between wire and brackets. These forces can result in decreased treatment efficiency, loss of anchorage, and consequently unwanted tooth movement.[3] Two major types of friction can be defined as static frictional force which is the smallest force needed to start a motion of solid surface, and kinetic frictional force which is the force required to resist the sliding motion of one solid object over another at a constant speed. Most studies use the term friction as a static frictional force that equals coefficient of frictional force multiply by forces perpendicular to line of motion (perpendicular to wire).[4] During sliding mechanism in orthodontic treatment, a part of the applied force is dissipated to overcome friction, while friction is transmitted to the tooth supporting structure inducing tooth movement.[5] It was reported that 12% to 60 % of the force induced by fixed orthodontic appliances are used to overcome friction.[6] Combinations of mechanical and chemical factors are determinant of friction between wires and brackets.[7] Some studies have investigated the factors associated with friction between wires and brackets and have listed them as clearance between wires and brackets, size of wire, cross section of the wire (round or rectangular), incorporated torque in bracket and wire, area of cross section of wire and bracket slot, wire and brackets materials, width of bracket slot, type of bracket (conventional versus self-ligate), type and amount of ligation force,[8] in addition to environmental condition such as temperature and presence of lubricant.[9] Each intermediate material that reduces contact area between two surfaces can be used as a lubricant or antifriction substance.[10] There are lots of controversies about the role of saliva in friction. Some studies have mentioned that saliva can reduce friction, while others express the opposite.[10] So far, few studies have assessed the effect of natural saliva in reducing or increasing the friction, and most of them have used artificial saliva in their investigations.[11] Friction in clinical orthodontics is now receiving more attention because orthodontic companies have proclaimed that low friction was good, and the concept was applied for marketing their self-ligating brackets.[12] The Damon SL bracket is a self-ligating bracket which does not exert spring pressure on the arch wire, and uses covers which slide vertically in an occlusal direction.[10] The slot size of these brackets is 0.022×0.027 inch^2^.[1] Nowadays, clear self-ligating brackets have become very popular in orthodontic practice and both the patients and orthodontists are more interested in using them. Since there is not enough evidence in regard to frictional forces in these types of brackets, the present study was designed. The aim of this study is to investigate and compare the amount of force resistance to sliding (combination of frictional force, binding, and notching)[12] in metallic and clear conventional MBT and Damon brackets in wet condition (natural saliva). To the best of authors’ knowledge, no study has been performed on clear Damon or self-ligating brackets, so far.

## Materials and Method

The study was done in Hamadan School of Dentistry and Amirkabir University. We used Damon 3MX bracket, Damon clear (Ormco Corporation; 1332 S Lone Hill Ave, Glendora, CA 91740, United States), as well as metallic and clear conventional brackets (FORESTADENT® USA; 2315 Weldon Parkway St. Louis, MO 63146, USA). Clear brackets did not have metallic slot and metallic brackets were made with injection molding technique in this study. The brackets had 0.022 inch slot pertaining to upper right canine; this bracket was chosen because canine teeth retract independently in non-en masse retraction. A 0.019×0.025 inch^2^ stainless steel (SS) wire (Ormco Corporation; 1332 S Lone Hill Ave, Glendora, CA 91740, United States) was used for sliding, because it is the main wire for sliding in Damon and other straight wire conventional (MBT) system. From each type of bracket, 10 brackets were investigated, so the samples included 40 brackets.

**Figure 1:**
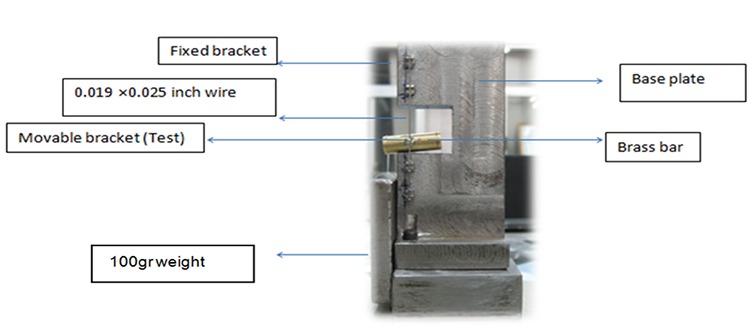
Combination of base plate, brackets, archwire, and the suspended 100g weight

A modification of Tidy approach[13] was used in this study (Figure 1).

**Figure 2:**
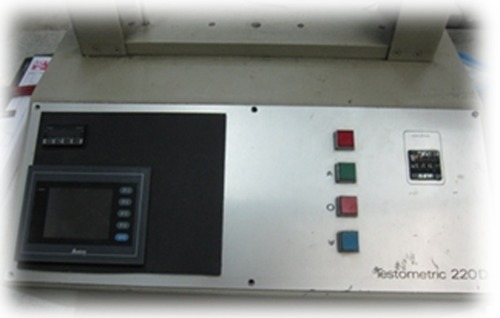
Testometric machine 220 D

The device consisted of a metallic base plate in which four conventional brackets were bonded with 8 mm distance. On the middle part of the fixed brackets, there was a 16mm space, and a movable bracket was positioned there. This bracket could slide along the arch wire. A 0.019×0.025 SS wire was passed through these brackets. The conventional brackets that were fixed to base plate were tied with ligature wire, while movable brackets were tied according to their self-ligating system and conventional brackets were tied with ligature wire. Oral cavity condition was simulated with un-stimulated saliva of the operator who had orthodontic appliance in his mouth. Saliva was applied with dropper to arch wire for each examination. The movable bracket was attached to the center of a brass bar with a cyanoacrylate and a 100 g weight was hung at a 10 mm distance from the center of bracket in order to represent equivalent single force acting on the resistance center of the tooth. The entire test was done using Testometric machine (220 D; Testometric co., unit 1, Lancashire, UK) with a cross head speed of 5 mm/min (Figure 2). The movable bracket was hung to the load cell throughout the test.

It is necessary to mention that before performing each examination and hanging the weights, the machine was calibrated to ignore the weight of movable bracket and brass bar. In each examination, after suspending the weights, movable brackets passed a distance more than 10 mm and the load cell recorded the force value. The force value recorded with load cell can be defined as the clinical force required for retracting a canine, while the difference between this value and the weights can be defined as a frictional force. The measurements and information from examinations were converted to graph using a software (Testometric’s feature-rich winTest™ Analysis software). In the graph, the first peak represents the static frictional force, and 7mm after the peak represents the value of resistance to sliding. Mean and standard deviation were measured for each bracket. Two-way ANOVA test was used for statistical analysis with respect to the type of brackets (clear versus metallic) and ligation (self-ligating versus conventional). The level of significant was set at *p*< 0.05.

## Results

**Figure 3:**
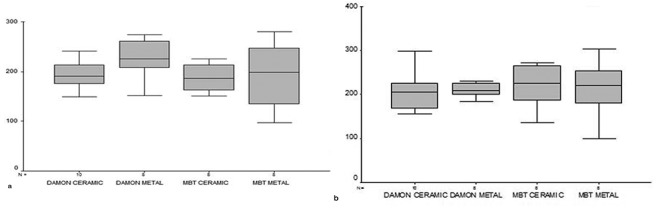
a: Mean resistance to sliding in the clear and metallic brackets of each type  b: Mean static frictional forces in the ceramic and metallic brackets of each type

The mean (±SD) of resistance to sliding was 194.88 (±26.65) and 226.62(±39.9) g for the esthetic and metallic Damon brackets, while these values were 187.81(±27.84) and 191.17(±66.68) g for the clear and metallic conventional brackets, respectively. Static frictional forces were 206.4(±42.45) and 210.38(±15.89) g in the esthetic and metallic Damon brackets and 220.63(±49.29) and 215.13(±62.38) in the clear and metallic conventional brackets. According to the two-way ANOVA test, no significant difference was seen between the two bracket materials (clear and metallic) and the two types of bracket (self-ligating versus conventional) in resistance to sliding (*p*= 0.17 and *p*= 0.23, respectively), and in static frictional forces (*p*= 0.55 and *p*= 0.96, respectively). No significant difference existed between esthetic and metallic brackets of conventional and Damon system regarding resistance to sliding (*p*= 0.36) and static friction (*p*= 0.77) (Figures 3).

## Discussion

Our finding indicate that the mean (±SD) of resistance to sliding was 226.62(±39.9)g for metallic Damon and 191.17 (±66.68)g for conventional brackets; while static frictional forces for metallic Damon was 210.38(±15.89)g and for conventional brackets, it was 215.13(±62.38)g. With respect to statistical analysis, bracket material (ceramic versus metallic) had no effect on resistance to sliding and static friction in the current study. One of the reasons may be using injection molding technique in manufacturing the clear brackets that were used in this study. It has been reported that this technique could decrease the frictional forces.[14]

Clocheret *et al.* in their study used a different combination of wire and brackets to measure coefficient of friction. They concluded although some of the ceramic brackets had slightly higher coefficient of friction, they still remain statistically comparable to stainless steel brackets.[15] This finding is somehow similar to that of the present study. It can be concluded that there is no contradiction in using ceramic brackets because for higher coefficient of friction when appropriate material selection and evaluation have been performed.

Nishio *et al.* in 2004,[16] Kusy *et al.* in 1990,[17] Karamonzos *et al.* in 1997,[18] Tanne *et al.* in 1991,[14] and Griffith *et al.* in 2005[19] reported the ceramic brackets had higher frictional forces than metallic ones; which is in contrast with the results of the present study. The differences in protocols of measuring frictional forces and the size of wire used in those studies and the current one might be the reasons for this conflict.

We performed the comparison only on the SS wire with the size of 0.019×0.025 inch^2^ because it was the recommended wire for sliding and space closure in both techniques. Regarding the wire size, majority of the studies have concluded that frictional forces were greater in ceramic brackets than the metallic ones in most wire sizes.[14, 16-18] However, it seems that with increasing the wire size and therefore decreasing the clearance, the difference between low-friction system and high-friction system would decrease. As concluded in the study of Tecco *et al.*, low-friction ligatures show lower friction compared with conventional ligatures when coupled with round archwires, but not with rectangular ones.[20] It can be another reason for our findings regarding the friction in metallic and ceramic brackets.

In this study, two-way ANOVA test revealed no significant difference between the two types of bracket (self-ligating versus conventional) regarding resistance to sliding (*p*= 0.23) and static frictional forces (*p*= 0.96).

The studies[4, 10, 21-25] can be divided into two groups based on the resistance force to sliding and static friction in Damon and conventional brackets. The first group[21-24] reported no difference between self-ligating and conventional brackets, while the second group[4, 10, 25] claimed that self-ligating brackets produce less friction than conventional ones.

Pandis *et al.* in 2007 mentioned there was no difference in frictional forces between self-ligating (Damon 2) and conventional brackets.[21] Scott *et al.* in 2008 compared conventional and self-ligating system in correction of dental irregularities. In systems, 0.014 NiTi, 0.014×0.025, 0.018×0.025 NiTi and 0.019×0.025 SS were used, orderly. The results of study showed that the rate of tooth movement had no significant difference in self-ligating and conventional brackets.[22] In a systematic review, Ehsani *et al.* concluded that until 2009, there had been only a little information to show that self-ligating brackets produce less friction than the conventional ones in the presence of rectangular wire with tipping and torque and in an arch with a severe malocclusion.[23] Krishnan *et al.* in 2009,[24] reported that using a 0.019×0.025 stainless steel wire made no difference in frictional forces in Damon and conventional brackets, which has probably been due to the use of wires that fill the slot of brackets. This can minimize the difference between Damon and conventional brackets, as it did in the present study. The results of these studies were similar to those of the current one.

The second group of studies claimed that self-ligating brackets produce less friction than conventional brackets.[4, 10, 25] The results of these studies were different from those of the present study. Henao and Kusy[25] used typodont as a model with different malocclusion. In their study the wire was pulled from the brackets and the drawing force values were used for analysis of friction. Pizzoni *et al.*[4] and Thomas *et al.*[10] used rigid bar with one fixed bracket, and pulled the wire from the bracket. They also used the drawing force for friction analysis. As it is known, this drawing force is combination of the force required to move the wire and the frictional forces. In our study, a removable bracket was used that slid along the wire; furthermore, a weight was hung at a 10 mm distance from the center of bracket to represent equivalent single force acting on the resistance center of the tooth. This design was more similar to the real sliding mechanism used in clinical practice. In the present study, unlike the above mentioned studies, the precise friction value can be identified by subtracting the weight value from the value obtained with testing machine. As mentioned by Clocheret *et al.*, most researchers have used different protocols or even approaches to evaluate the friction generated in different wire–bracket combinations.[15] Thus, the published results of many studies are difficult to compare. Using an *in-vitro* environment was probably a major limitation of this study; hence, these results should be used with caution due to the apparent difference between oral and *in-vitro* environment.

## Conclusion

There was no significant difference between clear and metallic Damon and conventional brackets regarding resistance to sliding and static friction, in wet condition on a 0.019×0.025 SS wire. In full- size archwires, there was a small difference between various bracket types and materials. 
